# Supplementary material for: MetaRibo-Seq measures translation in microbiomes
Source: Nat Commun. 2020 Jun 29;11:3268. doi: 10.1038/s41467-020-17081-z (PMC7324362; doi:10.1038/s41467-020-17081-z)
Supplement: Supplementary file 10 — Supplementary Data 7 [file 41467_2020_17081_MOESM10_ESM.zip › File2/Confidence_VeryHigh_Taxonomy/6046_out.krona.html]

Javascript must be enabled to view this page.

members
magnitude
magnitudeUnassigned
count
unassigned
taxon
rank

6046\_out

4

4
2
superkingdom

phylum
1
1224

class
1
28216

order
1
80840

family
119060
1

genus
47670
1

species
47671

SRS016474\_contig\_number\_9628
1

phylum
201174
3

3
1760
class

order
3
2037

family
2049
3

genus
1

SRS022143\_contig\_number\_contig-100\_6203.74122
3
1654

species
1

SRS147386\_contig\_number\_contig-100\_9689.9690
1655

species
1
544580

SRS015692\_contig\_number\_17129
